# Supplementary material for: Selective dorsal rhizotomy; evidence on cost-effectiveness from England
Source: PLoS One. 2020 Aug 10;15(8):e0236783. doi: 10.1371/journal.pone.0236783 (PMC7416930; doi:10.1371/journal.pone.0236783)
Supplement: S1 File — (DOCX) [file pone.0236783.s001.docx]

# Supplementary Material

## Imputation of missing SDR CtE data

We investigated predictors of missingness and predictors of outcomes (GMFM-66 and CPQOL-pain) in the SDR CtE data. We included CPQOL feelings about functioning domain score, gait profile (collected at baseline and 24 months only) as well as CPQOL-pain and GMFM-66 measured at baseline, 6, 12 and 24 months in an imputation model to predict missing data. We also included centre; age at assessment; GMFCS level; time from assessment to surgery; time to 6, 12 and 24 month follow-ups; using a wheelchair at 24 months(Y/N); and able to move independently at 24 months (Y/N). GMFM-66 and CPQOL domain scores at each follow-up point were included in the imputation model as the change in score from baseline. Missing data were less than 10% for all measures except gait analysis scores. We undertook 10 imputations and used Predictive Mean Matching for all variables.

## Assessment of incremental change in GMFM-66 following SDR

We used the growth curve models published for the CanChild cohort to predict for each child in the CtE cohort at two year follow-up, GMFM-66 if the child had not had SDR.(1,2) In summary, the CanChild cohort included 657 children with CP recruited over the period 1995-2001. Recruitment aimed for equal numbers in each GMFCS level. Children were excluded if they had received SDR, intrathecal baclofen or botulinum toxin. GMFM-66 was measured every 6-12 months with an average of 4 observations per child. Data were grouped within GMFCS level and modelled using nonlinear mixed-effects modelling. The CanChild researchers found a two parameter ‘stable limit’ model provided the best fit to data on children in GMFCS II for whom a decline in GMFM-66 in later adolescence was not observed. Data from children in GMFCS III fit best to a three parameter ‘peak/decline’ model in which GMFM-66 begins to fall around age 8 years.(2)

We used the stable limit model to predict GMFM-66 in the absence of surgery for children in GMFCS II (equation 1). The model has two parameters: a rate parameter, *λ*, governing the shape of the growth curve and a parameter θ for the maximum attained GMFM-66 score. We assumed a common rate parameter for all children in GMFCS II, taken from the published value. We used age and GMFM-66 at baseline to calculate the maximum GMFM-66 score for each child. We then calculated the predicted GMFM-66 score at the date of the two-year follow-up based on age at follow-up, the common rate parameter and the maximum GMFM-66 calculated for each child.

Stable limit model: *ŷ* = θ(1 – exp[-*λX*]) (equation 1)

where *y* is GMFM score, *X* is age, *λ* is the rate of change in GMFM-66 and θ is the maximum GMFM-66 attained.

We used the peak/decline model to predict GMFM-66 in the absence of surgery for children in GMFCS III (equation 2). This model has three parameters; *Y*_0_, the predicted GMFM-66 at age 6 years (72 months) serves a similar role to θ in the 2-parameter model. The remaining two parameters *δ* and *γ* influence the shape of the curve. We assumed *δ* was constant across children in GMFCS III, taken from the published value. We further assumed a constant ratio *Y*_0_/*γ* of 1.08017, calculated from the published values for *Y_0_* and *γ*. On this basis we were able to calculate *Y*_0_ and *γ* for each child using age and GMFM-66 at baseline. We then calculated the predicted GMFM-66 at the date of two-year follow-up according to the child’s age at follow-up.

Peak/decline model: *ŷ* = [ -*δ* (1 – *X*/*X*_0_) + *X*/*X*_0_.(*Y*_0_ - *δ*)/*γ* *^X^*^0^]*γ* *^X^* + *δ* (equation 2)

where *y* is GMFM score, *X* is age (months), *X*_0_ is 72, *Y*_0_ is the GMFM-66 at age *X*_0_ (72 months), and *δ* and *γ* are parameters influencing the shape of the curve.

We then calculated an incremental effectiveness of SDR on GMFM-66 for each child as the difference between the GMFM-66 observed at two years (or imputed) and that predicted using the relevant growth curve model. The incremental effectiveness of SDR on CPQOL-pain was calculated as the difference in the observed (or imputed) value at two-year follow-up and the value at baseline (prior to surgery).

## Quantifying uncertainty in outcomes

A two-stage bootstrapping routine was used to quantify the impact of SDR on GMFM-66 and CPQOL-pain at 24 months to reflect the clustering of data within centres.(3) The routine resamples at the cluster (centre) level first and then at the individual level. A ‘shrinkage’ correction is applied to avoid overestimating variance in samples with a small number of clusters. Separate bootstrapping routines were applied for GMFM-66 and CPQOL-pain, and undertaken on each of the ten imputed datasets. Bias corrected mean treatment effects and standard errors were combined across the ten imputations using Rubin’s rules to estimate the mean treatment effect and the standard error. In subgroup analysis, the process was applied to the respective subgroup of patients at GMFCS level II or level III.

## Imputation of missing cost data

Missing data were extensive beyond 5 years follow-up for the cohort of children in the RJAH cohort not receiving SDR. Data were imputed for each year with missing data. We undertook 20 imputations using Predictive Mean Matching. Due to limitations imposed by the number of observations we included only age at assessment and treatment arm (SDR/not SDR) in the imputation routine in addition to cost for each year of follow-up.

## Analysis of cost data

Table 1, below, lists the number of children receiving treatment according to the type of procedure for children in the group receiving SDR and the group that did not over the first five years of observation. The numbers in brackets give the number of procedures performed as some children received more than one procedure during a single surgical episode. Histograms of total cost data by treatment (SDR/not SDR) at five and ten years supported an assumption that the data was not strongly skewed. The histogram of costs at five years is shown in figure 1 below. Therefore, linear regression was used to quantify the incremental cost of SDR with adjustment for age and GMFCS level. In the sensitivity analysis we also adjusted for clinical criteria contraindicating SDR.

The incremental cost of SDR was calculated as the difference in mean costs over five years from assessment for SDR between children who received SDR and children who did not after controlling for differences in age and GMFCS category. In two sensitivity analyses we extended follow-up to ten years from assessment, and we included a dummy variable in the regression analysis to identify children declined SDR for reasons other than purely financial.

## Cost-effectiveness analysis

The ICER for SDR with respect to GMFM-66 was calculated as the incremental cost of SDR divided by the mean incremental effectiveness (observed minus predicted GMFM-66 score). Likewise, the ICER for SDR with respect to CPQOL-pain was calculated as the incremental cost of SDR divided by the mean incremental effectiveness (two year follow-up minus baseline CPQOL-pain score).

We undertook probabilistic analysis to quantify the impact of uncertainty in costs and outcomes of SDR and reported the results as Cost-Effectiveness Acceptability Curves (CEACs). The curves plot the likelihood that the incremental cost of SDR is outweighed by the value of the incremental benefit, dependent on the value placed on the health gain. We simulated estimates of the incremental cost of SDR over five years by specifying the incremental cost as a Normal distribution with mean and standard deviation derived from combining the results of linear regression across the 20 imputed datasets using Rubin’s rules. Similarly, we simulated estimates of the incremental gain in GMFM-66 of SDR at 24 months by specifying the treatment effect on GMFM-66 score as a Normal distribution with mean and standard deviation informed by combining the bootstrapped estimates of the mean and standard error of the treatment effect across each of the imputed dataset. One thousand random draws for the incremental costs were paired at random with 1,000 draws for the incremental gain in GMFM-66.

We calculated the proportion of the 1,000 pairs for which the Incremental Net Monetary Benefit (INMB) was positive at a threshold value of zero for a unit gain in GMFM-66 according to equation 3 below. The calculation was repeated assuming an increasing value on a unit gain in GMFM-66 in increments of £100 to a maximum of £5,000. The data were used to plot the CEAC for GMFM-66 (Figure 2 in the main text); the CEAC shows the proportion of simulations in which the INMB is positive (intervention is cost-effective) as the threshold willingness-to-pay for a unit gain in GMFM-66 increases from zero to £5,000.

INMB = *ΔE*λ* –*ΔC* (equation 3)

Where *ΔE* is the difference in GMFM-66, *ΔC* is the difference in cost and *λ* is the value placed on a unit change in GMFM-66.

The same approach was used to construct CEACs for the secondary outcome measure and the subgroup analyses. In each analysis we retained the original simulated incremental cost estimates, but paired them with simulated incremental gains in effectiveness for the relevant outcome and population group.

## Subgroup analysis results

The mean (SD) incremental gain in GMFM-66 is 6.1 (0.87) in GMFCS level II children and 4.6 (0.62) in level III children, respectively. The mean (SD) difference between CPQOL-pain dimension scores at baseline and 24 months is 11.0 (2.83) in GMFCS level II children and 6.0 (2.43) in level III children, respectively. Figures 2 and 3 report the CEACs for each subgroup with respect to the primary and secondary outcome and for the base case analysis and the sensitivity analysis controlling for clinical criteria contraindicating SDR.

|  | Number of children receiving treatment | |
| --- | --- | --- |
| Procedure | SDR (n = 15) | No SDR (n = 11) |
| SDR | 15 | 0 |
| Removal of metalwork | 0 | 3 (5) |
| Derotation osteotomies (tibial or femoral) | 4 (6) | 5 (9) |
| Calf release | 2 (4) | 3 (6) |
| Other soft tissue procedures | 1 | 1 (2) |
| Subtalar arthrodesis | 1 | 0 |
| Inpatient physiotherapy | 11 | 13 |
| Serial casting | 6 | 18 |
| Phenol to obturator nerve | 0 | 2 |
| Botox injection | 3 | 12 |

The numbers in brackets report the number of procedures (some patients underwent multiple procedures in the same surgery).

Table 1S. Procedures received by patients in the RJAH cohort

# Figure

Figure 1. Costs at five years for children in the RJAH cohort


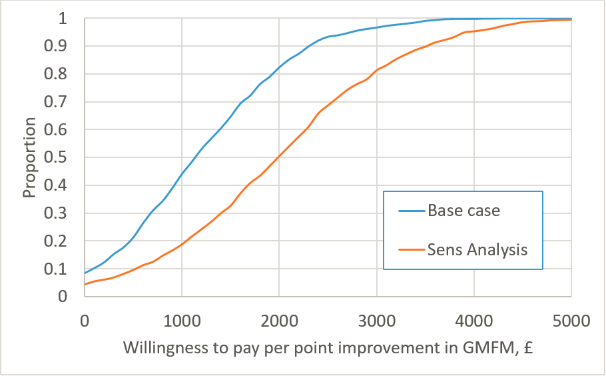


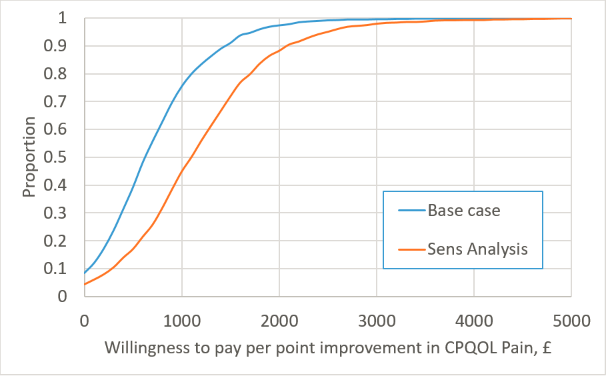


Figure 2. Cost-effectiveness acceptability curves for GMFM-66 and CPQoL-pain in subgroup GMFCS group II patients.


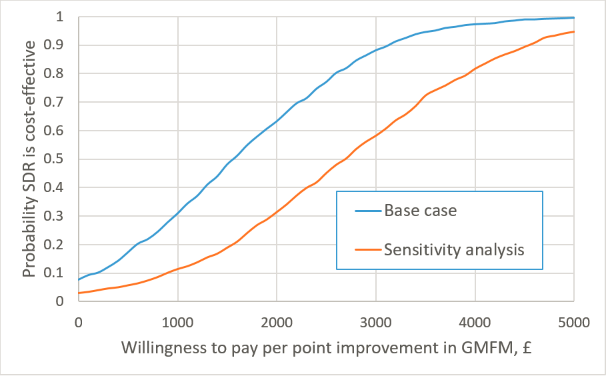


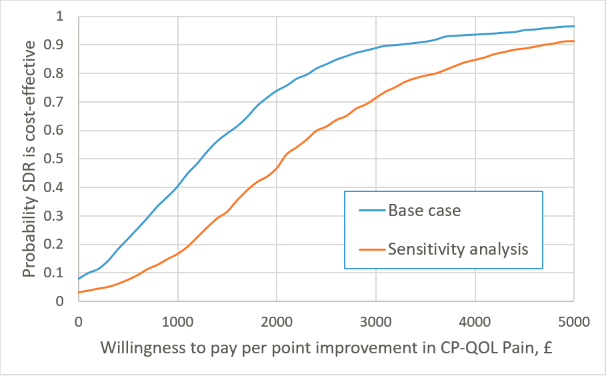


Figure 3. Cost-effectiveness acceptability curves for GMFM-66 and CPQoL-pain in subgroup GMFCS group III patients.

## References

1. Rosenbaum PL, Walter SD, Hanna SE, Palisano RJ, Russell DJ, Raina P, Wood E, Bartlett DJ, Galuppi BE. Prognosis for gross motor function in cerebral palsy: creation of motor development curves. JAMA. 2002 Sep 18;288(11):1357-63.

2. Hanna SE, Rosenbaum PL, Bartlett DJ, Palisano RJ, Walter SD, Avery L, Russell DJ. Stability and decline in gross motor function among children and youth with cerebral palsy aged 2 to 21 years. Developmental Medicine & Child Neurology. 2009 Apr;51(4):295-302.

3. Ng ES, Grieve R, Carpenter JR. Two-stage non-parametric bootstrap sampling with shrinkage correction for clustered data. Stata J. 2013 Jan 1;13(1):141-64.
